# Predictive Factors of Therapy-Related Cardiovascular Events in Patients with Lymphoma Receiving Anthracyclines

**DOI:** 10.3390/medsci12020023

**Published:** 2024-04-24

**Authors:** Alberto Lopez-Garcia, Ester Macia, Sandra Gomez-Talavera, Eva Castillo, Daniel Morillo, Jose Tuñon, Borja Ibañez, Raul Cordoba

**Affiliations:** 1 Lymphoma Unit, Department of Hematology, Fundación Jiménez Díaz University Hospital, IIS-FJD Health Research Institute, Avenida Reyes Catolicos, 228040 Madrid, Spain; 2 Cardio-Oncology Unit, Department of Cardiology, Fundacion Jimenez Diaz University Hospital, Avenida Reyes Catolicos, 228040 Madrid, Spain; 3 Centro Nacional de Investigaciones Cardiovasculares Carlos III (CNIC), 28029 Madrid, Spain; 4 CIBER de Enfermedades Cardiovasculares (CIBERCV), 28029 Madrid, Spain; 5 Department of Pharmacy, Fundación Jimenez Diaz University Hospital, Avenida Reyes Católicos, 228040 Madrid, Spain

**Keywords:** lymphoma, anthracyclines, cardiotoxicity, cardio-oncology

## Abstract

Background: Cancer-therapy-related cardiac dysfunction (CTRCD) is a growing concern for public health, with a growing incidence due to improved survival rates of patients with hematological malignancies due to diagnostic and therapeutic advances. The identification of patients at risk for CTRCD is vital to developing preventive strategies. Methods: A single-center retrospective cohort study was conducted between 1 January 2017 and 15 February 2023. Medical records of patients with lymphoma treated with first-line anthracyclines were reviewed. Demographic data, cardiovascular risk factors, biomarkers of myocardial damage, and echocardiographic information were collected. Results: A total of 200 patients were included. The incidence of CTRCD was 17.4% (35/200). Patients with CTRCD were older than those without CTRCD, with a mean age of 65.17 years vs. 56.77 (*p* = 0.008). Dyslipidemia (DL) (31.4% vs. 13.4% *p* = 0.017) and previous cardiovascular disease (40% vs. 13.3%; *p* < 0.001) were more frequent in the group who developed an event. Mean baseline NT-proBNP levels in the subgroup with cardiovascular events were 388.73 kg/L ± 101.02, and they were 251.518 kg/L ± 26.22 in those who did not (*p* = 0.004). Differences in Troponin I levels were identified during and after treatment without exceeding the laboratory’s upper reference limit. Patients were followed for a median of 51.83 months (0.76–73.49). The presence of a CTCRD event had a negative impact on overall mortality from any cause (HR = 2.23 (95% CI: 1.08–2.93); *p* = 0.031). Conclusions: Early identification of risk factors is crucial to manage patients at risk for CTRCD.

## 1. Background

Improved survival rates for patients with hematological malignancies due to diagnostic and therapeutic advances have led to an increase in the number of survivors at risk for long-term side effects of chemotherapy [1]. With an overall 5-year survival rate of around 70%, non-Hodgkin lymphoma (NHL) represents the seventh most frequent tumor worldwide, accounting for more than 4% of all cancers in the USA alone, with approximately 75,000 new cases per year, and a median age at diagnosis of 67 years [2,3]. In contrast, Hodgkin lymphoma (HL) presents overall 5-year survival rates of around 90%and is less common, with around 80% of cases diagnosed in patients younger than 65 years [4].

Anthracyclines have been a mainstay of treatment for both NHL and HL since 1976, when they were first used as part of different immunochemotherapy regimens, including cyclophosphamide, doxorubicin, vincristine, and prednisone (CHOP), before being combined with the anti-CD20 monoclonal antibody rituximab in 1995. Long-term cardiotoxicity, such as heart failure (HF), is a well-described side effect of treatment with anthracyclines used in regimens with curative intent [5], although other molecules used in lymphoma treatment (such as cyclophosphamide) are also capable of causing cardiotoxicity within the first days of treatment, leading to early HF [6].

The classic model of anthracycline-induced toxicity involves the hyperproduction of reactive oxygen species (ROS) and reactive nitrogen species (RNS), producing DNA damage, protein carbonylation, and lipid peroxidation and leading to cellular dysfunction and cardiomyocyte death. On the other hand, anthracyclines can also bind and block the functions of both topoisomerases 2A (TOP2A) and 2B (TOP2B). Tumor cells express high levels of TOP2A, whereas TOP2B is expressed ubiquitously, including by cardiomyocytes; this difference in topoisomerase expression leads to CRTCD [7].

Both the antitumor effect of anthracyclines and the probability of drug-related toxicity are dose-dependent. A lifetime cumulative dose of 400 mg/m^2^ has been associated with a 10% risk of developing congestive HF, while higher doses lead to an exponential increase in risk (up to 50% at 700 mg/m^2^) [8,9]. However, patient vulnerability to CTRCD is highly variable, with some patients developing HF at lower doses or long after ending chemotherapy.

The clinical spectrum of anthracycline CTRCD ranges from asymptomatic left ventricular systolic disfunction to advanced-stage HF with massive pump failure, potentially ending in heart transplantation or even death [10]. Other cardiotoxic events include myocardial infarction, thromboembolism, conduction disorders, valvopathies, and cardiomyopathies [11]. Although the mechanisms behind these events are not fully understood, patients with previous cardiovascular comorbidities (hypertension, left ventricular hypertrophy, diabetes, alcohol intake, smoking, etc.) have a higher risk of developing CRTCD [12].

Despite anthracycline-induced CRCTD being a well-described phenomenon, the use of anthracyclines as a first-line treatment for patients with NHL is expected to increase [13]. Careful assessment of the risk–benefit tradeoff between inducing HF and curing cancer is critical for both patients and health systems [10]. Our study aims to characterize patients with a high risk for anthracycline-associated CRCTD in order to improve the clinical management of these patients.

## 2. Material and Methods

### 2.1. Participants and Study Metods

We performed a retrospective study of patients diagnosed with NHL or HL and treated with anthracyclines as first-line therapy between 1 January 2017 and 31 December 2019. Patients were identified using the hospital pharmacy department’s database, which prospectively collects data on patients receiving anthracycline-containing regimens. Findings were compared with data from the electronic medical record. The follow-up period ended on15 February 2023.

### 2.2. Inclusion, Exclusion Criteria, and Study Period

All patients from the hospital pharmacy department’s database aged 18 years or above with a diagnosis of HL or NHL and who had received anthracycline-containing regimens as first-line therapy (R-CHOP, CHOP, R-mini-CHOP, mini-CHOP, R-COMP and R-ABVD, ABVD) were included. Patients who did not meet the main inclusion criteria were excluded. The anthracycline doses used were standard according to each chemotherapy protocol, and patients who had received concurrent therapy such as radiotherapy for lymphoma treatment were not included. Accumulated doses of anthracyclines were not recorded.

The baseline for the study period was immediately prior to starting a chemotherapy regimen with anthracyclines. Patients were followed until their last recorded appointment or death (until 15 February 2023).

### 2.3. Exposure and Covariates

A retrospective chart review, using data from the electronic medical record and the hospital pharmacy database, was carried out for all patients. We extracted treatment-related data, including chemotherapy regimen (R-CHOP, R-mini-CHOP, CHOP, mini-CHOP, R-COMP, ABVD, R-ABVD) and start date as well as lymphoma subtype (HL, diffuse large B cell lymphoma, follicular lymphoma, mantle cell lymphoma, mediastinal lymphoma, MALT lymphoma, and marginal zone splenic lymphoma).

Data on potential confounding variables were also collected, including patient demographics (age, sex, and BMI), cardiovascular risk factors (hypertension, dyslipidemia, alcohol intake, and smoking status), and previous medical history of cardiomyopathy, myopericarditis, or arrhythmias (defined as previous cardiovascular disease). Information on cardiovascular medication was also retrieved.

Analytical (hemoglobin, pro-BNP, troponin) and echocardiographic (left ventricle ejection fraction (LVEF) and presence of valve disease or cardiomyopathy) covariates were collected. Troponin levels were measured in relation to anthracycline administration as biochemical monitoring protocols before each anthracycline administration, as well as pro-BNP levels.

### 2.4. Outcomes

The primary objective of our study was to identify the incidence of CTRCD in patients with NHL or HL in first-line treatment with anthracyclines. Our secondary objective was to identify the prevalence of risk factors previously described in the literature in our cohort. Overall survival (OS) and time to treatment event (TTE) were also included as secondary endpoints.

We defined CTRCD as a composite of heart failure, myocardial infraction, thromboembolism, conduction disorders, and the development of valve disease or cardiomyopathy. This inclusive approach aims to capture the full spectrum of anthracycline-induced cardiac effects, acknowledging that while some events, like valve disease and thromboembolism, are less commonly attributed directly to anthracycline, their inclusion reflects the complex clinical presentations observed in practice.

### 2.5. Data Collection, Storage, and Ethics

We designed a database in Excel format, pseudonymizing patient data by assignation of a numerical identification code following the order of recruitment. The database did not contain patients’ personal information.

The raw data obtained during the study are considered confidential and will be treated in accordance with the General Data Protection Regulation (25 May 2016), which has been transposed into our legislation as the Organic Law 3/2018 (5 December 2018) on the protection of personal data. The database will be kept for up to two years after the end of the study, after which the data will be deleted permanently.

This study was conducted in accordance with the ethics standards of the institutional research committee and the Declaration of Helsinki, Hospital Clinical Research Ethics Committee (EO047-19_FJD). Being a retrospective study, it was not necessary to request informed consent.

### 2.6. Stastical Analysis

A descriptive analysis of the population was performed. Qualitative variables were described using frequency tables. Continuous variables that followed a normal distribution were presented as the mean and standard deviation. Continuous variables that did not follow a normal distribution were presented as the median and interquartile range.

Normality was verified using the Kolmogorov–Smirnov test. To study the relationship between the different variables, Student’s *t* tests or analysis of variance were used for variables with normal distribution, and the Mann–Whitney or Kruskal–Wallis tests were used for variables with non-normal distribution. Univariate analyses were carried out, and those variables with statistically significant results were entered into a multivariate regression model. Survival curves were traced via the Kaplan–Meier method and compared with the logarithm of the rank (LogRank) test. Statistical analyses were performed using SPSS v25 for Windows (SPSS Inc., Chicago, IL, USA).

## 3. Results

A total of 200 patients were included. Incidence of CTRCD and cardiovascular risk factors were identified by collecting data from participants’ electronic medical records. Patients’ characteristics are shown in Table 1.

The incidence of CTRCD was 17.4% (35/200). Patients suffering from CTRCD were older, with a mean age of 65.17 ± 16.41 years vs. 56.77 ± 17.18 years in the subgroup of patients who did not develop CT (*p* = 0.008). The distribution of cardiotoxic events is shown in Table 2.

DL was more frequent in the group who developed an event (36.84% vs. 16.67%; *p* ≤ 0.00001), as well as previous cardiovascular disease (61.05% vs. 14.02%; *p* ≤ 0.00001). We found no differences between other cardiovascular risk factors described in the literature such as female sex (21.54% vs. 18.75%), hypertension (30.3% vs. 16.67%), diabetes (20% vs. 20.17%), obesity (21.15% vs. 19.84%), smoking (21.43% vs. 19.54%), or alcohol intake (13% vs. 18.8%) (Table 3).

We found no differences between patients receiving liposomal anthracyclines (all of whom presented previous cardiovascular disease) and those receiving conventional therapy (27.8% vs. 17%; *p* = ns). Likewise, no differences in cardiotoxic events were found between patients with and without previous reduced LVEF (14.3% vs. 18.1%; *p* = ns (Table 4)) or with and without valve disease (21.5% vs. 15%; *p* = ns). However, we found significant differences between patients with and without previous history of cardiomyopathy at diagnosis (24.5% vs. 11.8%; *p* = 0.026).

Prescriptions of cardiovascular drugs, such as angiotensin converting enzyme (ACE) inhibitors, beta-blockers (BB), or angiotensin receptor antagonists (ARA), were recorded as part of the data collection process. These prescriptions formed part of patients’ habitual treatment for underlying cardiovascular disease, with no differences in CTRCD incidence between groups.

Regarding cardiac biomarkers of myocardial infarction, no differences in troponin values were observed at baseline, although differences were found at intermediate (0.031–0.035 µg/L; *p* = 0.0009) and final (post-treatment) assessment (0.007–0.033 µg/L; *p* = 0.0082). Although these troponin values do not exceed our laboratory’s upper reference limit (N < 0.08 µg/L), differences between values reach statistical significance, suggesting that in our cohort, an increase in troponin levels during treatment could be considered a risk factor for developing CTRCD (Table 5).

Mean baseline NT-proBNP levels in the group of patients with cardiovascular events were 456.1 µg/L ± 101.02, and 120.1 µg/L ± 26.22 in the “no event” group (*p* = 0.052). Mean NT-proBNP at mid-treatment was 536.5 µg/L ± 111.03 vs. 125.9 µg/L ± 31.78 (*p* = 0.03), while mean NT-proBNP after completion of treatment was 785.7 µg/L ± 678.76 vs. 165.4 µg/L ± 37.88 (*p* = 0.033). The upper reference limit in our laboratory is 250 µg/L (Table 6).

No significant differences in hemoglobin levels were observed between groups at baseline and during follow-up, although a tendency towards lower hemoglobin counts was found in the cardiotoxic event group before, during, and after treatment. Mean baseline hemoglobin was 11.8 gr/dL ± 0.34 for patients with a cardiotoxic event and 12.6 gr/dL ± 0.16 in those without (*p* = 0.503) (Table 7).

After performing the univariate analysis and identifying statistically significant variables, we conducted a multivariate analysis (Table 8), identifying dyslipidemia, age 72 years or older, previous cardiovascular disease, and basal NT-proBNP as risk factors for cardiotoxicity in patients receiving treatment with anthracyclines as a first-line treatment for lymphoma.

**Figure 1:**
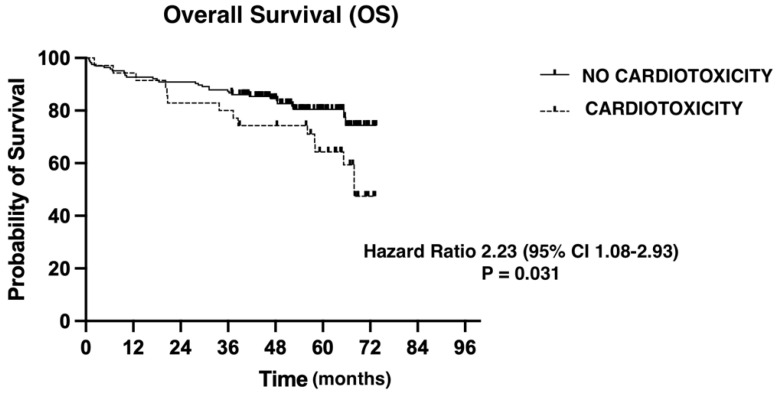
The impact of cardiotoxicity on overall survival.

We evaluated overall survival using the Kaplan–Maier method (Figure 1). Median follow-up was 51.83 months (0.76–73.49). The presence of a CTRCD event had a negative impact on OS from any cause (hazard ratio = 2.23 (95% CI: 1.08–2.93); *p* = 0.031).

**Figure 2:**
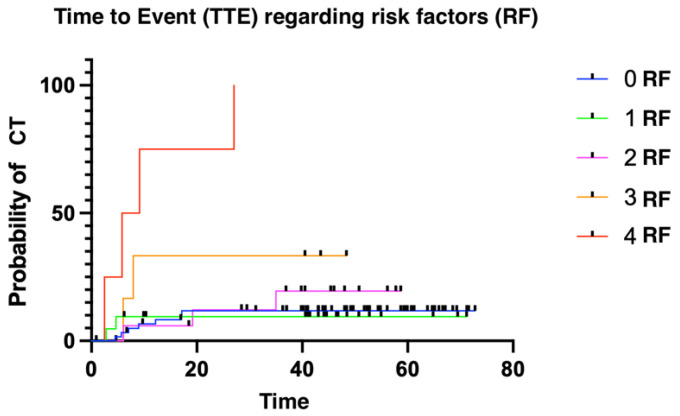
Time to event according to risk factors.

**Figure 3:**
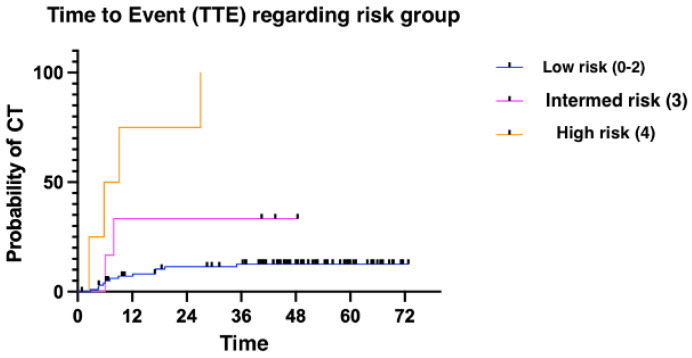
Time to event according to risk group.

**Figure 4:**
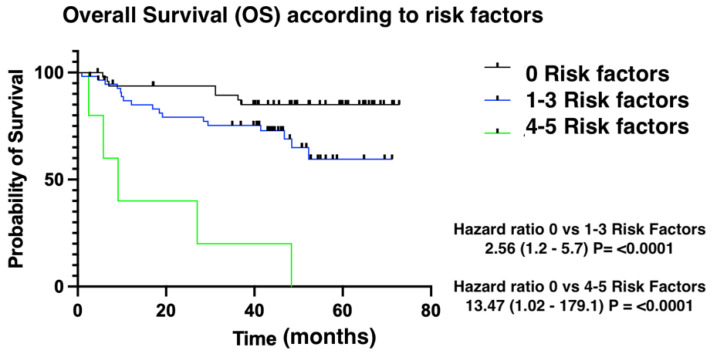
Overall survival according to risk factors.

The median time to presenting CTRCD was 6.11 months (1.02–52.86). We divided patients into three groups: low risk (≤two risk factors for CT); intermediate risk (three risk factors); and high risk (four risk factors). Patients presenting more risk factors for cardiotoxicity presented with cardiotoxic events earlier than patients with fewer risk factors (Figure 2 and Figure 3). Finally, overall survival was also influenced by the number of risk factors for CTRCD. In this case, low-risk patients were identified as those with no risk factors, intermediate-risk patients as those with between one and three risk factors, and finally, high-risk patients as those with four risk factors (Figure 4).

## 4. Discussion

Anthracycline-induced cardiotoxicity is a growing challenge for public health due to an ageing population with an increasing prevalence of cardiovascular risk factors and the widespread use of anthracyclines in lymphoma therapy, with high response rates and improved overall survival [14]. Our study reports incidence and risk factors for cardiotoxicity in a cohort of 200 lymphoma patients undergoing first-line treatment with anthracyclines.

The incidence of cardiotoxicity in our cohort is 17.4%, higher than that found in previous studies. Boddicker et al. report cardiotoxicity rates of 10.7% [15], while Curigliano et al. describe an incidence of 3–5% [16]. This difference may be due to several factors. First, the aforementioned studies do not include venous thrombosis in their definition of a cardiotoxic event [17]. Secondly, discrepancy exists between different echocardiographic criteria for cardiotoxicity [10]. Our institution uses the classification published by the American Society of Echocardiography (ASE) and the European Association of Cardiovascular Imaging (EACVI), which defines cardiotoxicity as a decrease in left ventricular ejection fraction (LVEF) of more than 10 percentage points below the normal reference value (53%), independently of the presence or absence of symptoms [18].

Incorporating microangiopathy could significantly enhance our understanding of its mechanisms, especially in light of findings from the AIM PILOT study which suggests a critical role of microcirculation disorders in anthracycline-induced cardiotoxicity. Furthermore, observations around liposomal anthracyclines offer intriguing insights into their potential protective effects against CTRCD, warranting further investigation. Highlighting these aspects not only broadens the discourse on cardiotoxicity but also underscores the importance of continued research into protective strategies and the underlying pathophysiology of CTRCD [19].

Age has been reported as a risk factor for CTRCD. Our study reports statistically significant differences in the mean age of patients with and without CT. Aging is associated with aortic stiffness, left ventricle remodeling due to myocardial hypertrophy, myocardial fibrosis, and cardiac amyloidosis. Increased susceptibility to toxicity is determined by complex interactions between the cardiovascular aging process and cardiovascular risk factors, comorbidities (such as anemia or kidney disease), and disease modifiers such as sex [20].

Several cardioprotective interventions using classical HF drug therapies (beta-blockers, angiotensin-converting enzyme inhibitors, and angiotensin II receptor blockers) have recently been tested in controlled trials, although none have shown robust beneficial effects to date. Although these studies have many limitations, including important heterogeneity, some indicate a potential clinical benefit for the prevention of HF [20,21]. In our cohort, however, patients taking classical HF drug therapies did not present a lower risk of CT, although it is important to highlight that the medication formed part of chronic treatment for underlying PCVD instead of primary prevention.

Regarding echocardiography, generally speaking, only patients with normal LVEF are prescribed anthracyclines, pointing to a selection bias in our cohort as only patients with normal LVEF were selected to receive anthracyclines. This same bias occurred in patients treated with liposomal anthracyclines as only patients with previous cardiac disorders were prescribed this variation, aimed at reducing the risk of CTRCD, probably underestimating its protective factor for the development of this toxicity [22]. Of the biomarkers of myocardial damage featured in our cohort, only NT-proBNP proved a risk factor for cardiotoxicity in the multivariate analysis. Troponin I levels were significantly different between groups during and after treatment, although not at baseline, with mean values within the laboratory’s normal reference limits.

It is vital to align with the 2022 European Society of Cardiology (ESC) guidelines, which introduce comprehensive criteria for diagnosing cardiotoxicity. These guidelines emphasize the importance of left ventricular ejection fraction (LVEF) and global longitudinal strain (GLS) as critical parameters. LVEF, a traditional measure of cardiac function, and GLS, a sensitive indicator of subtle myocardial deformation, together provide a robust framework for early detection and diagnosis of cancer-therapy-related cardiac dysfunction (CTRCD), ensuring timely intervention and management.

An important finding of our study is that the number of risk factors for cardiotoxicity impacts both time to treatment event as well as overall survival related to death from any cause. This emphasizes the importance of closely monitoring lymphoma patients receiving anthracycline therapy and implementing strategies for optimal management of risk factors such as dyslipidemia and previous cardiovascular disease.

Our study is limited by its retrospective design, leading to missing information on the control of cardiovascular risk factors during treatment. Also, our analysis did not consider variables that may directly influence plasma levels of biomarkers for myocardial damage. The influence of factors such as renal failure or obesity on troponin I plasma levels has been described in the literature and may be a confounding factor in our study [23,24].

Another relevant caveat of our study is the dosage of anthracyclines. Although it was administered in a routine manner following the immunotherapy protocols previously described, the total cumulative dose was not accounted for. This omission is critical since the cumulative effect of anthracyclines, which has been extensively studied, shows a negative impact at higher doses over the patient’s lifetime.

This retrospective study highlights a well-known aspect. On one hand, it identifies a special risk population for the development of CTRCD, on the other hand, the high incidence and its impact on the survival of these patients. Therefore, new primary prevention strategies are necessary in order to improve the prognosis of this population.

In this regard, multiple pharmacological and non-pharmacological strategies are being developed. One of the most promising is remote ischemic preconditioning (RIPC), which has shown clinical benefits in porcine models and is currently being tested in humans in the RESILIENCE clinical trial. This trial includes, as described in this patient cohort, high-risk patients for the development of CTRCD [25].

Cardio-oncology is a complex field which requires individual patient assessment as well as the creation of multidisciplinary teams. Where future developments are concerned, the recent publication of the European Society of Cardiology’s guideline on cardio-oncology [25] and the application of techniques such as cardiac magnetic resonance imaging in oncology patients [26] will help to identify patients at risk of developing anthracycline-associated cardiotoxicity in a more homogeneous and precise manner, allowing clinicians to develop effective preventive strategies.

## 5. Conclusions

Our study demonstrates that age is a risk factor for anthracycline-induced cardiotoxicity. Dyslipidemia, previous cardiovascular disease, and increased NT-proBNP levels at baseline and follow-up are also predictors of cardiotoxicity. On the other hand, reduced LVEF was not associated with an increased risk of CTRCD, probably due to the positive selection of patients with normal ventricular function at the start of treatment. Patients with a previous history of cardiomyopathy presented a higher incidence of cardiotoxicity, while no differences were found in patients with valve disease 

In our cohort, the use of cardioprotective drugs was not associated with reduced rates of cardiotoxicity. A possible explanation is the existence of a certain selection bias, as the drugs had been prescribed to treat underlying conditions instead of as primary prevention. Selection bias may also explain why patients with normal baseline LVEF and those receiving liposomal anthracyclines showed similar rates of cardiotoxicity to those with reduced LVEF and those receiving standard anthracycline treatment.

Patients who developed CTRCD during treatment with anthracyclines presented higher all-cause mortality rates than patients without cardiotoxic events. The number of risk factors is correlated with time to event, as well as with overall survival.

CTRCD is an important challenge for cardio-oncologists worldwide. Actions geared towards reducing modifiable risk factors such as dyslipidemia and optimizing management of cardiovascular comorbidities are key to preventing cardiotoxic events in patients with lymphoma.

## Figures and Tables

**Table 1:** Study population characteristics.

Median Age (Range)	59 (18–89)
Sex (male)	101 (50.2%)
LYMPHOMA SUBTYPE	
Diffuse large B lymphoma	86 (42.9%)
Hodgkin lymphoma	54 (27%)
Follicular lymphoma	38 (19%)
Primary mediastinal B cell lymphoma	10 (5.1%)
Non-Hodgkin lymphoma T	7 (3.6%)
Mantle cell lymphoma	5 (2.6%)
REGIMEN USED	
R-CHOP	103 (51.7%)
ABVD	51 (25.4%)
RminiCHOP	21 (10.4%)
R-DAEPOCH	15 (7.5%)
BEACOPP	4 (2%)
CHOEP	3 (1.5%)
VR-CAP	3 (1.5%)
CARDIOVASCULAR RISK FACTORS	
Hypertension (HT)	50 (25%)
Diabetes (DM)	13 (6.5%)
Dyslipidemia (DL)	51 (25.5%)
Previous cardiovascular disease	35 (17.5%)
Obesity	65 (32.8%)
Alcohol intake	24 (12%)
Smoker or former smoker	60 (30.5%)

R-CHOP: rituximab, cyclophosphamide, doxorubicin, vincristine, and prednisone; ABVD: doxorubicin, bleomycin, vinblastine, and dacarbazine; R-DAEPOCH: etoposide, prednisone, vincristine, cyclophosphamide, doxorubicin, and rituximab; BEACOPP: Bleomycin, etoposide, doxorubicin, cyclophosphamide, vincristine, procarbazine, prednisolone; CHOEP: cyclophosphamide, doxorubicin, vincristine, ethopoxide, and prednisone; VR-CAP: bortezomib, rituximab, cyclophosphamide, doxorrubicin, prednisone.

**Table 2:** CTRCD events (n = 35).

Heart Failure	12 (34.4%)
Myocardiopathy	9 (25.7%)
Thromboembolism	7 (20%)
Myocardial Infarction	3 (8.5%)
Conduction disorders	3 (8.5%)
Valve disease	1 (2.9%)

**Table 3:** Cardiovascular risk factors and risk of CTRCD.

	Event (CT)	No Event (NO CT)	*p*-Value
**Median age (CI 95%)**	70.85 y/o (65.3–76.39)	54.62 y/o (51.23–58.01)	*p* < 0.0001
Hypertension (HT)	30.30%	16.67%	*p* 0.09
Dyslipidemia (DL)	36.84%	13.19%	*p* 0.037
Diabetes	20%	20.17%	*p* 0.98
Smoker or former smoker	21.43%	19.54%	*p* 0.8
Alcohol intake	13%	18.8%	*p* 0.67
Obesity	21.15%	19.84%	*p* 0.81
Sex	21.54%	18.75%	*p* 0.83
Previous cardiovascular disease	61.05%	14.02%	*p* < 0.0001

**Table 4:** LVEF and cardiotoxicity.

Baseline LVEF			
	Event	No Event	
AVERAGE	61.15%	60.59%	
CI 95%	58.85–63.46%	59.35–61.83%	*p* 0.92

LVEF: Left ventricular ejection fraction.

**Table 5:** Troponin I levels.

Troponin I (N < 0.08 µg/L)			
	Event	No Event	
BASELINE TROPONIN			
AVERAGE	0.022 µg/L	0.018 µg/L	
IC 95%	0.013–0.031 µg/L	0.014–0.022 µg/L	*p* 0.23
INTERMEDIATE TROPONIN			
AVERAGE	0.031 µg/L	0.025 µg/L	
IC 95%	0.021–0.041 µg/L	0.01–0.04 µg/L	*p* 0.0009
FINAL TROPONIN			
AVERAGE	0.07 µg/L	0.033 µg/L	
IC 95%	0.024–0.1 µg/L	0.026–0.039 µg/L	*p* 0.0082

**Table 6:** NT proBNP levels.

NT-PROBNP			
	Event	No Event	*p*-Value
BASELINE NT-proBNP			
AVERAGE	456.1 µg/L	120.1 µg/L	
CI 95%	156.2–755.9 µg/L	87.4–152.9 µg/L	*p* 0.0052
INTERMEDIATE NT-proBNP			
AVERAGE	563.5 µg/L	125.9 µg/L	
CI 95%	223.9–903.1 µg/L	94.05–157.8 µg/L	*p* 0.03
FINAL NT-proBNP			
AVERAGE	785.7 µg/L	165.4 µg/L	
CI 95%	208.9–1363 µg/L	124.1–206.7 µg/L	*p* 0.022

NT-proBNP: N-terminal B-type natriuretic peptide.

**Table 7:** Results of hemoglobin levels.

	Event	No Event	
BASAL HEMOGLOBIN			
AVERAGE	11.8 gr/dL	12.6 gr/dL	
CI 95%	10.87–12.47 gr/dL	11.25–16.41 gr/dL	*p* 0.503
INTERMEDIATE HEMOGLOBIN			
AVERAGE	11.28 gr/dL	11.96 gr/dL	
CI 95%	10.81–11.75 gr/dL	11.66–12.27 gr/dL	*p* 0.3
FINAL HEMOGLOBIN			
AVERAGE	11.52 gr/dL	11.94 gr/dL	
CI 95%	10.74–12.3 gr/dL	11.6–12.27 gr/dL	*p* 0.24

**Table 8:** Multivariate analysis.

	*p*-Value	Odds Ratio (OR) CI 95%
Dyslipidemia	0.05	3.18 (1.45–6.71)
≥72 years	0.047	3.79 (1.78–8.1)
PCVD	0.018	4.57 (2.02–10.35)
Basal NT-proBNP	0.02	17.5 (3.1–99.7)

## Data Availability

The participants of this study did not give written consent for their data to be shared publicly, so due to the sensitive nature of the research supporting data is not available.

## List of Abbreviations

ABVD: doxorubicin, bleomycin, vinblastine, and dacarbazine
ACE: angiotensin-converting enzyme
ARBs: angiotensin II receptor blockers
ASE: American Society of Echocardiography
BEACOPP: bleomycin, etoposide, doxorubicin, cyclophosphamide, vincristine, procarbazina, and prednisolone
CHOEP: cyclophosphamide, doxorubicin, vincristine, ethopoxide, and prednisone
CT: cardiotoxicity CTRCD: cancer-therapy-related cardiac dysfunction CI: confidence interval
DL: dyslipidemia
EACVI: European Association of Cardiovascular Imaging
HF: heart failure HT: hypertension
HL: Hodgkin lymphoma
LV: left ventricular
LVEF: left ventricular ejection fraction
MALT: mucosa-associated lymphoid tissue
NHL: non-Hodgkin lymphoma
NT PROBNP: N-terminal B-type natriuretic peptide OR: odds ratio OS: overall survival PCVD: previous cardiovascular disease
R-CHOP: rituximab, cyclophosphamide, doxorubicin, vincristine, and prednisone
R-DAEPOCH: etoposide, prednisone, vincristine, cyclophosphamide, doxorubicin, and rituximab
RNS: reactive nitrogen species
ROS: reactive oxygen Species
TOP2A: topoisomerases 2A
TOP2B: topoisomerases 2B TTE: time to event
VR-CAP: bortezomib, rituximab, cyclophosphamide, doxorrubicin, and prednisone
